# Analysis of Time to the Hospital and Ambulance Use Following a Stroke Community Education Intervention in China

**DOI:** 10.1001/jamanetworkopen.2022.12674

**Published:** 2022-05-17

**Authors:** Jing Yuan, Minghui Li, Yang Liu, Xiaomo Xiong, Zhengbao Zhu, Fangyu Liu, Yong Wang, Wei Hu, Z. Kevin Lu, Renyu Liu, Jing Zhao

**Affiliations:** 1 Department of Clinical Pharmacy, School of Pharmacy, Fudan University, Shanghai, China; 2 Department of Clinical Pharmacy and Translational Science, University of Tennessee Health Science Center, Memphis; 3 Department of Neurology, Minhang Hospital, Fudan University, Minhang District, Shanghai, China; 4 Clinical Pharmacy and Outcomes Sciences Department, University of South Carolina, Columbia; 5 Department of Epidemiology, School of Public Health, Medical College of Soochow University, Suzhou, China; 6 Department of Neurology, The First Affiliated Hospital of Chongqing Medical University, Chongqing, China; 7 Department of Cardiology, Minhang Hospital, Fudan University, Minhang District, Shanghai, China; 8 Department of Anesthesiology and Critical Care, Perelman School of Medicine at the University of Pennsylvania, Philadelphia; 9 Department of Neurology, Perelman School of Medicine at the University of Pennsylvania, Philadelphia

## Abstract

**Importance:**

Prehospital delay (time from symptom onset of stroke to the door of a hospital) in patients with stroke is long in China. With the goal of improving public awareness and knowledge of stroke recognition, Stroke 1-2-0 was developed in China as an education program to prompt rapid response to the onset of stroke based on clinical practice in China, and examination of its outcomes is needed.

**Objective:**

To investigate the association of the Stroke 1-2-0 educational campaign with prehospital delay for patients with ischemic stroke.

**Design, Setting, and Participants:**

In a population-based cross-sectional study, all patients with ischemic stroke events were admitted to the Minhang Hospital, which is the only tertiary care hospital with a stroke center that provides acute stroke care in Xinzhuang county, Shanghai, China. The study period was from January 1, 2016, to December 31, 2019, and data analysis was performed from January 1 to July 31, 2021.

**Exposures:**

A multifaceted Stroke 1-2-0 educational campaign comprising slides, videos, brochures, and posters distributed in the community.

**Main Outcomes and Measures:**

Proportion of patients with hospital arrival within 3 hours and use of an ambulance to seek medical care, as well as the odds of seeking medical attention within 3 hours after the stroke before vs after initiation of the multifaceted educational campaign.

**Results:**

A total of 2857 patients (1774 men [62.1%]; mean [SD] age, 69.83 [12.66] years) with stroke were identified, including 503 in the precampaign period and 2354 in the postcampaign period. Following the multifaceted campaign, the median (IQR) prehospital delay time decreased from 18.72 (7.44-27.84) hours to 6.00 (2.00-16.35) hours (*P* < .001). After the implementation of the Stroke 1-2-0 campaign, the proportion of patients with hospital arrival time within 3 hours increased from 5.8% to 33.4% (*P* < .001) and use of an ambulance increased from 3.2% to 30.6% (*P* < .001). In an interrupted time series analysis, the initiation of the Stroke 1-2-0 campaign was associated with significantly increased odds of arriving at the hospital within 3 hours (odds ratio, 8.01; 95% CI, 7.17-8.95; *P* < .001) and use of an ambulance (odds ratio, 9.41; 95% CI, 8.24-10.74; *P* < .001).

**Conclusions and Relevance:**

The persistent multifaceted campaign using the Stroke 1-2-0 program was associated with reduced prehospital delay and improved timely arrival rate and ambulance arrival rate for patients with stroke. These findings suggest that Stroke 1-2-0 can be adopted in other regions of China to possibly improve health outcomes and reduce clinical burdens for all patients with stroke.

## Key Points

**Question:**

Is Stroke 1-2-0, a community education campaign on rapid assessment of an individual potentially having a stroke, associated with time to arrival at the hospital and the use of an ambulance?

**Findings:**

This population-based cross-sectional study evaluated 2857 Chinese individuals with ischemic stroke. Following implementation of the Stroke 1-2-0 program, the delay in hospital arrival time was significantly decreased and use of an ambulance was significantly increased.

**Meaning:**

The findings of this study suggest a multifaceted campaign increasing the recognition of stroke and appropriate action may be useful in achieving timely hospital arrival.

## Teaser

This cross-sectional study examines hospital arrival time and use of ambulances following implementation of a community education program to increase recognition of stroke.

## Introduction

Stroke is a leading cause of death in China, resulting in more than 1.5 million deaths every year.^1^ The mortality rate of stroke in China is 5 times that of Europe and the US.^2^ Although the disability-adjusted life-years lost caused by stroke have decreased in the past 20 years in China, stroke is still associated with the highest loss of disability-adjusted life-years among all diseases.^3,4^ Stroke is an emergency and requires immediate medical attention. If a stroke episode is not followed by timely medical actions, patients will be at a major risk of death and disability.^5^ It is well documented in the literature that patients with stroke treated with thrombolytic therapy within 3 hours of the first symptom have a substantially lower disability rate compared with those who receive delayed treatment.^6,7,8^ Based on the current best estimate, the median hospital delay time for patients with stroke in China was nearly 15 hours, almost 5 times the recommended 3 hours.^9,10^

To create a mnemonic tool for rapid stroke recognition in China, the Stroke 1-2-0 program was developed by adapting the Face Arm Speech Time (FAST) program,^11,12^ which is a widely used tool designed for stroke recognition and rapid response to stroke onset.^13,14^ The emergency medical services telephone number 120 in China is transformed into 3 actions. In the Stroke 1-2-0 program, 1 refers to the first step of rapid stroke recognition to look at whether there is an asymmetrical face, 2 represents the second step to examine whether there is weakness in the arms, and 0 is pronounced the same as the phrase *listening carefully* in Chinese, which indicates listening closely to determine whether patients can speak clearly.^11,12^ If any of the symptoms in 1, 2, or 0 occur, the emergency medical services telephone number (120) should be dialed immediately. The Stroke 1-2-0 program has been recommended by the Chinese Stroke Association as a unique tool for stroke awareness education and has been implemented into their clinical guidelines for stroke.^12^

The first multifaceted campaign of the Stroke 1-2-0 program was launched in 2016 to improve public recognition of stroke.^15^ To our knowledge, no empirical population-based study has been conducted to examine the outcomes of Stroke 1-2-0 regarding response to stroke. To fill the gap in the literature, in this study, we aimed to examine the association between the Stroke 1-2-0 campaign and prehospital delay time (time from symptom onset of stroke to the door of a hospital) and timely arrival rate for patients with stroke at the population level, using time-series analysis.

## Methods

### Stroke 1-2-0 Multifaceted Campaign

The Stroke 1-2-0 program was promoted in Xinzhuang, Shanghai, through a multifaceted outreach campaign, which was developed based on the social ecological model.^16^ The multifaceted outreach program included broadcast videos, printed materials, local news, and face-to-face community education, targeting both patients and their environment (eg, family members) to improve their awareness of stroke symptoms. The multifaceted campaign was implemented from October 1, 2016, through December 31, 2019. More details about the campaign are described in the Box.

Box. **Description of the Multifaceted Stroke 1-2-0 Outreach Campaign Components**

Lectures by trained physicians every week
Television broadcast on local channel 6 times per day
Radio broadcast on local station 6 times per day
Print advertisement in local newspaper each month
Posters distributed to residential communities by trained project staff each month
Brochures distributed to residential communities by trained project staff each month

In brief, the Stroke 1-2-0 television campaign ran through free media advertising on the local TV channel, using a 1-minute animated video, which was endorsed by the Chinese Stroke Association and the special task force.^17^ The Stroke 1-2-0 video was broadcast 6 times per day. The public poster campaign was launched across Xinzhuang county, distributing approximately 100 000 brochures to residents during the campaign period. Approximately every household, or 1 in 3 residents, was exposed to at least 1 source of the information containing the Stroke 1-2-0 tool. Furthermore, face-to-face education sessions were offered by primary care physicians who were trained to use the Stroke 1-2-0 education materials, including a stroke training kit with slides, videos, brochures, and posters. During the campaign period, approximately 50 physicians delivered more than 200 education sessions.

### Study Design

This study used a population-based cross-sectional design of all patients with ischemic stroke admitted to the Minhang Hospital, which is the only tertiary care hospital with a stroke center in Xinzhuang county of Shanghai, China. Minhang Hospital has provided a routine thrombolysis service since 2015. An interrupted time series (ITS) analysis was used to retrospectively evaluate the association between the Stroke 1-2-0 campaign and time to the hospital.^18,19^ The precampaign Stroke 1-2-0 period was from January 1 to September 30, 2016; the program was implemented on October 1, 2016, and the postcampaign period was from November 1, 2016, to December 31, 2019. Data analysis for the present study was conducted from January 1 to July 31, 2021. This study was reviewed and approved by the ethics committees of the institutional review boards of Minhang Hospital of Fudan University. We followed the Strengthening the Reporting of Observational Studies in Epidemiology (STROBE) guideline for cohort studies.

### Data Source

Data were extracted anonymously from the hospital information systems. As a member of the China Stroke Center Alliance, which monitors the quality measures in stroke centers across the nation, Minhang Hospital routinely collected data for patients with stroke within 24 hours of admission. Data were abstracted into a standardized electronic database stored in the hospital information systems by trained personnel, in accordance with the data quality assurance process developed by the China Stroke Center Alliance. The data quality assurance processes included training of the medical staff in data collection, logic checks in the electronic data entry, and annual audits of random medical records to ensure high quality of the data. On presentation to the stroke center, patients or their caregivers were interviewed for their medical history and administered the National Institutes of Health Stroke Scale (NIHSS) (score 1-4 indicates minor stroke; 5-14, moderate stroke; ≥15, major stroke).^20^ The following data were retrieved from the electronic medical records, including age, sex, admission date and time, date and time of symptom onset, mode of transportation to the hospital, NIHSS score, and neurologic deficit.

### Case Identification

Patients who met the following criteria were included in the study: (1) had ischemic stroke confirmed with computed tomography or magnetic resonance imaging, (2) presented within 2 days of stroke onset, and (3) lived in Xinzhuang, Shanghai. To ensure the ascertainment of data on all patients with suspected ischemic stroke, we reviewed medical records from the emergency department to identify patients presenting with symptoms of ischemic stroke and reviewed death certificates to identify patients who were dead on arrival at the hospital.^2,13^ Patients were excluded if they (1) had missing data on age, sex, NIHSS score, and date and time of symptom onset; (2) had onset of ischemic stroke within the hospital; and (3) had dementia or psychological disorders. Patients with psychological disorders were excluded because they have abnormal thoughts, feelings, and behaviors and were unable to report the stroke onset accurately. We also performed sensitivity analyses to test the robustness of results.

### Outcome Measurement

Primary outcomes of interest included time to hospital and use of an ambulance. We also evaluated the proportion of patients who received tissue plasminogen activator (tPA). Time to hospital was defined as the time from the onset of stroke to arrival at the hospital and was categorized as within 3 hours and within 24 hours. These 2 cutoff thresholds were clinically relevant to emergency stroke treatment. We collected the time of stroke onset from patients or their caregivers who noticed stroke-related symptoms. If the symptoms occurred during the night or sleep, the time of awakening was used as the time of stroke onset.^21,22^ The time of hospital arrival and admission were recorded routinely for every patient who was admitted to the hospital. The use of an ambulance was based on the mode of transportation to the hospital that was reported by patients or their caregivers.^23^

### Statistical Analysis

To describe patient characteristics before and after the campaign, categorical variables are reported by frequencies with percentages and compared with χ^2^ tests. Continuous variables are described by median IQR and compared with the Mann-Whitney test. The proportion of hospital arrival time within 3 hours and within 24 hours was reported for each month and stratified by the stroke severity. The ITS analysis using a segmented logistic regression model was performed to evaluate the association between the Stroke 1-2-0 campaign and the proportion of patients arriving at the hospital within 3 or 24 hours and via ambulance in a given month. A logistic regression model was used to access the change in the log odds (slope) and intercept after the start of the Stroke 1-2-0 program.^24^ We also adjusted for potential confounding variables including age, sex, stroke severity, cigarette smoking, alcohol use, history of ischemic stroke or transient ischemic attack, comorbidities, and weekend or daytime onset. The Durbin-Watson statistics were used to test the autocorrelation of errors, and first-order serially correlated errors were corrected using the calendar month as a dummy variable when needed to control for seasonality (eTable 1 in the Supplement). All data analyses were conducted using SAS, version 9.4 (SAS Institute Inc). A 2-sided threshold of *P* < .05 was considered statistically significant.

## Results

### Patient Characteristics

**Figure 1.**
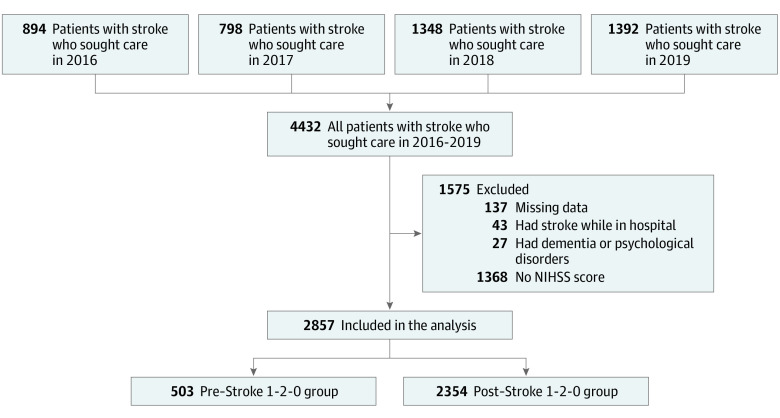
Sample Selection Process NIHSS indicates National Institutes of Health Stroke Scale.

A total of 503 patients with stroke (17.6%) were included in the precampaign period and 2354 (82.4%) patients with stroke were included in the postcampaign period. The population included 1774 men (62.1%) and 1083 women (37.9%); mean (SD) age was 69.83 (12.66) years. From January 1, 2016, to December 31, 2019, 4432 patients with confirmed ischemic stroke were admitted to Minhang Hospital. The study excluded 137 patients who had missing data on the time of stroke onset or arrival time at the hospital, 43 patients who had the onset of ischemic stroke while hospitalized, 27 patients who had dementia or psychological disorders, and 1368 patients who had missing data on the NIHSS score. Therefore, a total of 2857 patients with stroke were included in the analysis. Figure 1 outlines the sample selection process.

There were no significant differences between patients with stroke in the precampaign and postcampaign periods in age (mean [SD], 70.24 [12.81] vs 69.74 [12.63]; *P* = .43), alcohol use (60 [11.9%] vs 225 [9.6%]; *P* = .11), history of ischemic stroke or transient ischemic attack (113 [22.5%] vs 460 [19.5%]; *P* = .14), onset of stroke during the daytime (365 [72.6%] vs 1609 [68.4%]; *P* = .06), and onset of stroke during the weekend (143 [28.4%] vs 619 [26.3%]; *P* = .60) (Table 1). However, compared with the postcampaign period, the precampaign period had a greater proportion of those with moderate (151 [30.0%] vs 531 [22.6%]) and severe (25 [5.0%] vs 48 [2.0%]) (*P* < .001) stroke. The proportion of patients receiving tPA was significantly higher in the postcampaign period compared with the precampaign period (234 [9.9%] vs 28 [5.6%]; *P* < .001).

**Table 1.** Characteristics of Patients With Stroke

Characteristic	Before Stroke 1-2-0 (n = 503)	After Stroke 1-2-0 (n = 2354)	*P* value
Age, mean (SD), y	70.24 (12.81)	69.74 (12.63)	.43
Age range, y			
18-44	13 (2.58)	83 (3.53)	.49
45-64	159 (31.61)	688 (29.23)	
65-75	141 (28.03)	704 (29.91)	
>75	190 (37.77)	879 (37.34)	
Sex			
Female	211 (41.95)	872 (37.04)	.04
Male	292 (58.05)	1482 (62.96)	
NIHSS score^a^			
1-4	327 (65.01)	1775 (75.40)	<.001
5-14	151 (30.0)	531 (22.6)	
≥15	25 (5.0)	48 (2.0)	
Cigarette smoking	124 (24.65)	656 (27.87)	<.001
Alcohol use	60 (11.9)	225 (9.6)	.11
History of stroke or TIA	113 (22.5)	460 (19.5)	.14
Medical condition			
Hypertension	340 (67.59)	1544 (65.59)	.39
Diabetes	136 (27.04)	689 (29.27)	.32
Cerebral and/or subarachnoid hemorrhage	11 (2.19)	61 (2.59)	.60
Atrial fibrillation	45 (8.95)	164 (6.97)	.12
Daytime onset (6 AM to 6 PM)	365 (72.6)	1609 (68.4)	.06
Weekend onset	143 (28.43)	619 (26.30)	.60
Receiving tPA	28 (5.6)	234 (9.9)	<.001

Abbreviations: NIHSS, National Institutes of Health Stroke Scale; TIA, transient ischemic attack; tPA, tissue plasminogen activator.

^a^ Minor stroke is defined as NIHSS severity level score less than or equal to 4; moderate, 5 to 14; major, greater than or equal to 15.

### Time to Hospital

**Figure 2.**
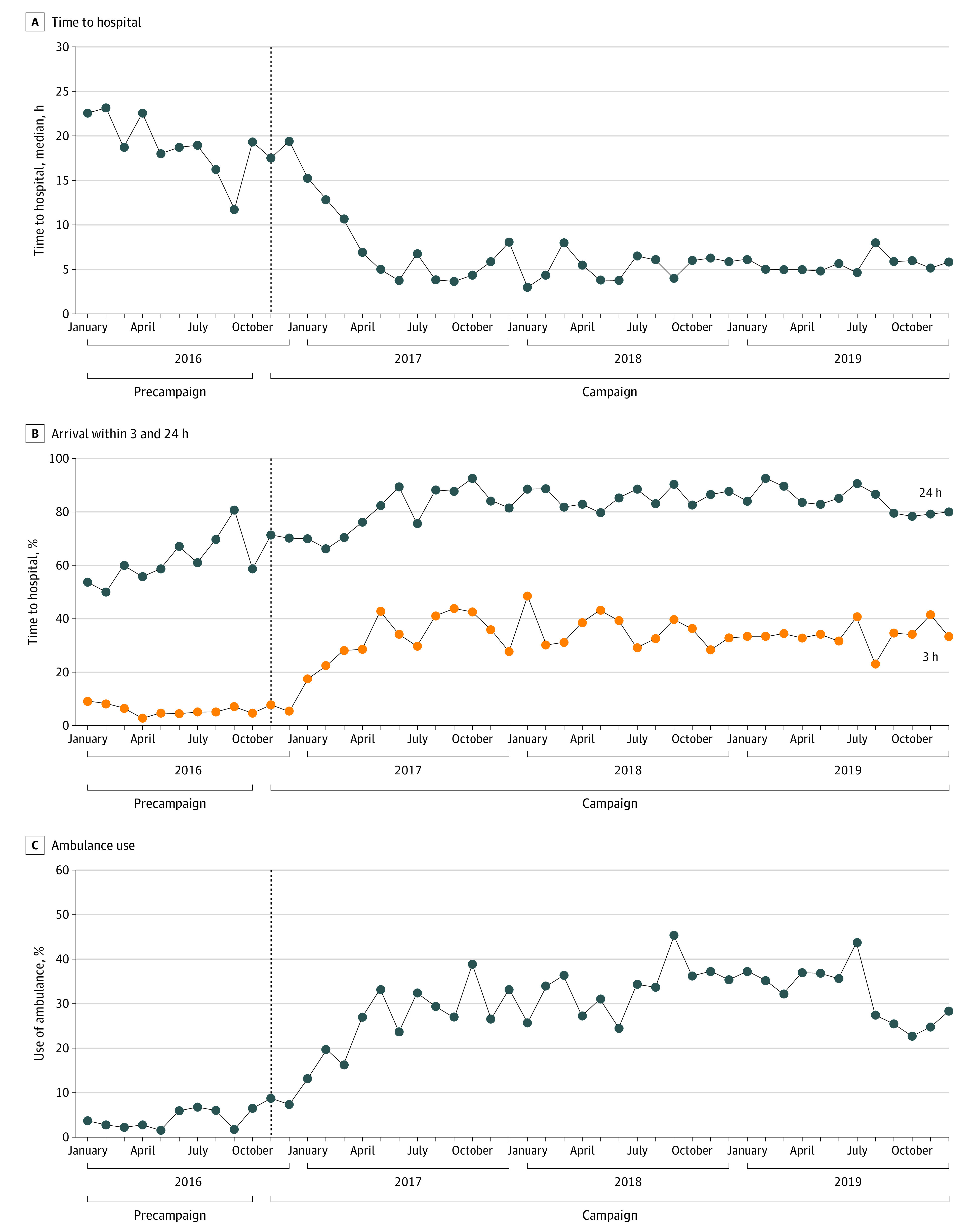
Hospital Data Before and After Implementation of the Stroke 1-2-0 Campaign

The median prehospital delay time decreased steeply after the implementation of the Stroke 1-2-0 campaign (Figure 2A). The median prehospital delay time was 18.72 hours (IQR, 7.44-27.84) before the Stroke 1-2-0 campaign and 6.00 hours (IQR, 2.00-16.35) after the Stroke 1-2-0 campaign (Table 2) (*P* < .001). There were significant decreases in the median time to hospital before and after the Stroke 1-2-0 campaign (Table 2) both for minor stroke (from 19.92 to 6.00 hours; *P* < .001) and for moderate or major stroke (from 15.96 to 5.00 hours; *P* < .001).

**Table 2.** Time to Hospital and Use of Ambulance Before and After Stroke 1-2-0 Campaign by Stroke Severity

Outcome	No. (%)		OR (95% CI)^a^
	Pre–Stroke 1-2-0	Post–Stroke 1-2-0	
**Overall**			
No.	503 (100)	2354 (100)	NA
Time to hospital			
Median (IQR), h	18.72 (7.44-27.84)	6.00 (2.00-16.35)	NA
≤3 h	29 (5.8)	787 (33.4)	8.01 (7.17-8.95)
≤24 h	312 (62.0)	1983 (84.2)	1.68 (1.58-1.79)
Use of ambulance	16 (3.2)	721 (30.6)	9.41 (8.24-10.74)
**Minor stroke^b^**			
No.	327 (65.0)	1775 (75.4)	NA
Time to hospital			
Median (IQR), h	19.92 (8.64-28.08)	6.00 (2.08-16.99)	NA
≤3 h	17 (5.2)	570 (32.1)	8.95 (7.80-10.28)
≤24 h	193 (59.0)	1479 (83.3)	1.74 (1.62-1.88)
Use of ambulance	10 (3.1)	456 (25.7)	10.47 (8.87-12.36)
**Moderate or major stroke**			
No.	176 (35.0)	579 (24.6)	NA
Time to hospital			
Median (IQR), h	15.96 (6.00-27.60)	5.00 (1.82-14.50)	NA
≤3 h	12 (6.8)	217 (37.5)	6.19 (5.11-7.50)
≤24 h	119 (67.6)	504 (87.0)	1.54 (1.38-1.72)
Use of ambulance	6 (3.4)	265 (45.8)	7.28 (5.80-9.14)

Abbreviations: NA, not applicable; NIHSS, National Institutes of Health Stroke Scale; OR, odds ratio.

^a^All differences significant at *P* < .001. In the interrupted time series analysis, use of a logistic regression model assessed the change in log odds of outcomes after implementation of the Stroke 1-2-0 campaign. Outcomes were the proportion of patients with time to hospital within 3 hours and within 24 hours, as well as the use of an ambulance. The covariates included cigarette smoking, diabetes, daytime onset of stroke, and use of an ambulance. The calendar month was included as a dummy variable to control for seasonality.

^b^ Minor stroke is defined as NIHSS severity level score less than or equal to 4; moderate, 5 to 14; major, greater than or equal to 15.

### Hospital Arrival Within 3 and 24 Hours

There were improvements in the proportion of patients with hospital arrival within 3 hours and within 24 hours after implementation of the Stroke 1-2-0 campaign (Figure 2B). The proportion of patients with hospital arrival time within 3 hours increased from 5.8% in the precampaign period to 33.4% in the postcampaign period and the proportion of patients with hospital arrival time within 24 hours increased from 62.0% to 84.2% (Table 2).

In the ITS analysis, there was a significant increase in the odds of arriving at the hospital within 3 hours from the end of the precampaign period to just after the beginning of the campaign (odds ratio [OR], 8.01; 95% CI, 7.17-8.95; *P* < .001), with an increasing trend after the campaign (OR, 1.08; 95% CI, 1.06-1.10; *P* < .001). The implementation of the Stroke 1-2-0 campaign was associated with increased odds of hospital arrival within 24 hours (OR, 1.68; 95% CI, 1.58-1.79; *P* < .001), with a downward trend after the campaign (OR, 0.92; 95% CI, 0.91-0.93; *P* = .001). Time to the hospital was particularly improved among those with major stroke and with the onset of stroke during the day but was less likely to improve among women, individuals who smoked cigarettes, those with a history of stroke or transient ischemic attack and those with atrial fibrillation (eFigure 1 in the Supplement).

For minor stroke, the proportion of patients with hospital arrival within 3 hours increased from 5.2% in the precampaign period to 32.1% in the postcampaign period (Table 2), and the percentage of patients with hospital arrival within 24 hours increased from 59.0% to 83.3%. The introduction of the Stroke 1-2-0 campaign was associated with increased odds of hospital arrival within 3 hours (OR, 8.95; 95% CI, 7.80-10.28; *P* < .001) and 24 hours (OR, 1.74; 95% CI, 1.62-1.88; *P* < .001).

For moderate and major stroke, the proportion of patients with hospital arrival within 3 hours increased from 6.8% to 37.5% between the precampaign and postcampaign periods, and the proportion with hospital arrival within 24 hours increased from 67.6% to 87.0%. In the ITS analysis, the odds of arriving at the hospital within 3 hours (OR, 6.19; 95% CI, 5.11-7.50; *P* < .001) and within 24 hours (OR, 1.54; 95% CI, 1.38-1.72; *P* = .001) were increased significantly from the end of the precampaign period to just after the beginning of the campaign.

### Use of an Ambulance

After the implementation of the Stroke 1-2-0 campaign, the percentage of patients with ambulance use increased substantially (Figure 2C). Overall ambulance use was 3.2% in the precampaign period and 30.6% in the postcampaign period (Table 2). In the ITS analysis, the odds of ambulance arrival increased significantly (OR, 9.41; 95% CI, 8.24-10.74; *P* < .001) from the end of the precampaign period to just after the beginning of the campaign. The odds of using an ambulance were increased significantly for patients with minor stroke (OR, 10.47; 95% CI, 8.87-12.36; *P* < .001) and moderate or major stroke (OR, 7.28; 95% CI, 5.80-9.14; *P* < .001) (eFigure 2 in the Supplement).

### Sensitivity Analysis

In the sensitivity analysis, after including patients without NIHSS scores, the Stroke 1-2-0 campaign demonstrated similar patient characteristics (eTable 2 in the Supplement) and an association between time to the hospital and use of an ambulance (eFigure 3 in the Supplement). In the sensitivity analysis using a 7-day time window in patient selection, there were upward trends in the time to the hospital and use of an ambulance, suggesting that our results were not sensitive to the 2-day time window (eFigure 4 in the Supplement).

## Discussion

In this population-based study, we found that time to the hospital for patients with stroke was significantly decreased following implementation of the Stroke 1-2-0 education campaign. The proportion of patients with stroke who received tPA was nearly doubled after the campaign. Therefore, these findings suggest that the Stroke 1-2-0 campaign is a promising way to improve emergency treatment of stroke and potentially improve a patient’s recovery from stroke.

Evidence has shown that using tPA in the early stage of stroke onset can significantly increase the odds of survival and independent function.^25,26^ A meta-analysis pooling data from 6 randomized clinical trials suggests that the earlier patients with stroke receive tPA, the better the health outcomes.^27^ However, owing to various reasons, such as transportation problems, unawareness of stroke, or lack of knowledge on the importance of timely stroke treatment, a prehospital delay has become the most critical limiting factor in the management of stroke, resulting in most patients with stroke being unable to receive tPA in time for the agent to be effective.^28,29^ Therefore, reducing time to hospital arrival and increasing the use of an ambulance are of great importance to improve the treatment outcomes for patients with acute stroke. In our study, the proportion of patients who arrived at the hospital within 3 hours and received tPA increased significantly after the Stroke 1-2-0 campaign, which could potentially improve patient outcomes.

However, this study found that, even after the implementation of the Stroke 1-2-0 campaign, the median time to hospital arrival was 6 hours, which was still twice the recommended 3 hours for effective use of tPA. Also, only one-third of patients with stroke arrived at the hospital within 3 hours of stroke onset. This percentage of timely arrival is much lower compared with that in other countries and regions.^30,31^ Therefore, development of innovative strategies to further expand the Stroke 1-2-0 campaign may be beneficial, particularly targeting individuals with higher risks of stroke. In the Houston Paramedic and Emergency Stroke Treatment and Outcomes Study, a multilevel education program was implemented to improve the accuracy of rapid hospitalization and diagnosis by nursing staff and increase the number of patients evaluated within the 3-hour window for use of tPA.^32^ There are other validated instruments for rapid stroke detection, including the Cincinnati Prehospital Stroke Scale and the Los Angeles Prehospital Stroke Screen in the US.^33,34^ Lessons can be learned from these different validation instruments and educational programs to further improve the design of Stroke 1-2-0.

Our findings suggest that stroke severity and patient characteristics were factors in the outcomes of the Stroke 1-2-0 campaign. More patients with mild stroke were admitted to the hospital after the campaign, suggesting patients and their family members were more alert to stroke symptoms and sought care even when the symptoms were mild. Time to hospital arrival was less likely to be improved among women or those with a history of stroke or transient ischemic attack, which could be explained by their probability of having some knowledge of stroke symptoms. Therefore, population characteristics and disease epidemiologic factors should be considered when developing local campaigns.

### Strengths and Limitations

This study has strengths. First, to our knowledge, this is the first empirical study in China to evaluate the association between the Stroke 1-2-0 campaign and the rates of prehospital delay, timely arrival, and ambulance use. Our findings provide clinical data for further promotion and support the use of Stroke 1-2-0. Second, our study included a subgroup analysis based on stroke severity. The results of this analysis suggest that the Stroke 1-2-0 campaign is useful for both minor and moderate or major strokes, compared with the FAST tool, which is less beneficial in patients with minor stroke.^23^ Third, our study used a sensitivity analysis, noting that the results were robust when including patients without NIHSS scores.

The study has limitations. First, the study collected data from only 1 hospital, potentially decreasing the generalizability of the findings. Second, this was a retrospective study using secondary data. Thus, some potential confounding variables were not collected. Prospective studies could consider incorporating more covariates in the form of surveys or clinical trials. Third, the study had a short precampaign period, beginning in January 2016, with the postcampaign period starting in November 2016. We have included a long follow-up period until the end of 2019 to examine the sustained and long-term outcomes of the Stroke 1-2-0 campaign. Fourth, the study evaluated only 1 link in a complex chain of patient care. In-hospital stroke care, including time to neuroimaging and transition of care, may also determine patient outcomes. Future studies should be conducted to assess the association of the campaign with treatment outcomes.

## Conclusions

Data from this study noted the persistent multifaceted Stroke 1-2-0 campaign was associated with reduced time to hospital arrival and improved use of tPA for patients with stroke, suggesting that the Stroke 1-2-0 campaign may potentially improve patient outcomes. Local adaptations of the Stroke 1-2-0 campaign are needed given the health disparities in urban and rural areas and economic development in different regions of China.

## Notes

Supplement. **eTable 1.** Durbin-Watson Statistics of Outcome Measures **eTable 2.** Additional Analysis of All Patients Who Were Admitted to the Hospital **eFigure 1.** Segmented Time-Series Regression Analysis for Time to Hospital ≤3 and ≤24 Hours **eFigure 2.** Segmented Time-Series Regression Analysis for Use of Ambulance **eFigure 3.** Sensitivity Analysis 1: Time to Hospital and Ambulance Use per Month Among All Stroke Patients (Regardless of NIHSS Score) **eFigure 4.** Sensitivity Analysis 2: Time to Hospital and Ambulance Use per Month With a 7-Day Time Window for Sample Selection
