# Supplementary material for: Analysis of Time to the Hospital and Ambulance Use Following a Stroke Community Education Intervention in China
Source: JAMA Netw Open. 2022 May 17;5(5):e2212674. doi: 10.1001/jamanetworkopen.2022.12674 (PMC9115614; doi:10.1001/jamanetworkopen.2022.12674)
Supplement: Supplement. — eTable 1. Durbin-Watson Statistics of Outcome Measures eTable 2. Additional Analysis of All Patients Who Were Admitted to the Hospital eFigure 1. Segmented Time-Series Regression Analysis for Time to Hospital ≤3 and ≤24 Hours eFigure 2. Segmented Time-Series Regression Analysis for Use of Ambulance eFigure 3. Sensitivity Analysis 1: Time to Hospital and Ambulance Use per Month Among All Stroke Patients (Regardless of NIHSS Score) eFigure 4. Sensitivity Analysis 2: Time to Hospital and Ambulance Use per Month With a 7-Day Time Window for Sample Selection [file jamanetwopen-e2212674-s001.pdf]

Supplemental Online Content

Yuan J, Li M, Liu Y, et al. Analysis of Time to the Hospital and Ambulance Use Following a Stroke Community Education Intervention in China. *JAMA Netw Open.* 2022;5(5):e2212674. doi:10.1001/jamanetworkopen.2022.12674

eTable 1. Durbin-Watson Statistics of Outcome Measures

eTable 2. Additional Analysis of All Patients Who Were Admitted to the Hospital

eFigure 1. Segmented Time-Series Regression Analysis for Time to Hospital ≤3 and ≤24 Hours

eFigure 2. Segmented Time-Series Regression Analysis for Use of Ambulance

eFigure 3. Sensitivity Analysis 1: Time to Hospital and Ambulance Use per Month Among All Stroke Patients (Regardless of NIHSS Score)

eFigure 4. Sensitivity Analysis 2: Time to Hospital and Ambulance Use per Month With a 7-Day Time Window for Sample Selection

This supplemental material has been provided by the authors to give readers additional information about their work.

© 2022 Yuan J et al. *JAMA Network Open*.

Supplemental file

eTable 1. Durbin-Watson statistics of outcome measures

	Durbin–Watson d statistic	Durbin's alternative test		
		Lag (0)	Lag (1)	Lag (2)
**3-hour arrival rate**	0.592	43.512 (P<.001)	1.723 (P=0.197)	1.786 (P=0.181)
**24-hour arrival rate**	0.793	21.952 (P<.001)	2.255 (P=0.141)	2.484 (P=0.096)
**Use of ambulance**	0.584	43.128 (P<.001)	0.275 (P= 0.603)	0.382 (P=0.685)

© 2022 Yuan J et al. *JAMA Network Open*.

eTable 2. Additional analysis of all patients who were admitted to the hospital (regardless of NIHSS score)

**Characteristics**	**Pre-Stroke 1-2-0 (n=695)**	**Post-Stroke 1-2-0 (n=3,530)**	***P*-value**
**Age**, mean (SD)	70.34 (±12.81)	69.96 (±12.89)	0.48
**Age**			0.49
18–44	19 (2.73%)	131 (3.71%)	
45–64	212 (30.50%)	1012 (28.67%)	
65–75	199 (28.63%)	1045 (29.60%)	
75+	265 (38.13%)	1342 (38.02%)	
**Female Sex**	286 (41.15%)	1365 (38.67%)	0.22
**Stroke severity (NIHSS)**			<.001
Minor	327 (47.05%)	1775(50.28%)	
Moderate/major	176 (25.33%)	579(16.40%)	
Unknown	192 (27.63%)	1176 (33.31%)	
**Cigarette smoker**	180 (25.90%)	862 (24.42%)	<.001
**Alcohol drinker**	86 (12.37%)	286 (8.10%)	<.001
**Prior stroke or TIA**	145 (20.86%)	585 (16.57%)	0.007
**Medical conditions**			
Hypertension	476 (68.49%)	2080 (58.92%)	<.001
Diabetes mellitus	181 (26.04%)	888 (25.16%)	0.62
CH&SH	15 (2.16%)	78 (2.21%)	0.93
Atrial Fibrillation	64 (9.21%)	240 (6.80%)	0.029
**Daytime onset**	489 (70.36%)	2423 (68.64%)	0.099
**Weekend onset**	196 (28.20%)	1107 (31.36%)	0.558

Abbreviation: SD, standard deviation; TIA, Transient ischemic attack; **CH**, cerebral hemorrhage; **SH**, subarachnoid hemorrhage; **NIHSS**, National Institutes of Health Stroke Scale; **PVD**, Peripheral Vascular Disease. Daytime was defined as 6AM to 6 PM.

© 2022 Yuan J et al. *JAMA Network Open*.

eFigure 1. Segmented time-series regression analysis for time to hospital ≤3h and ≤24h.

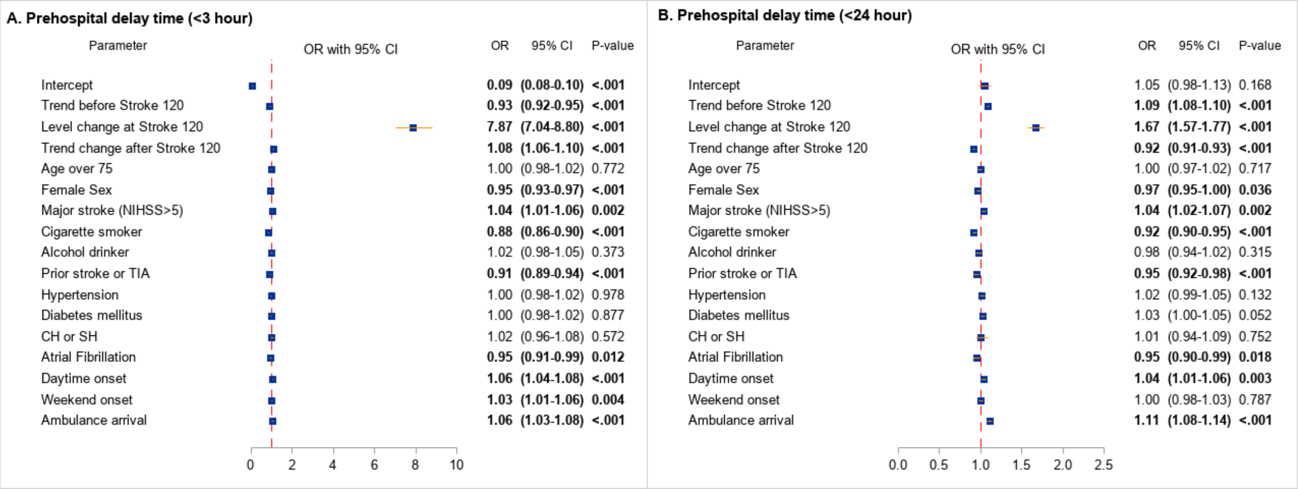

© 2022 Yuan J et al. *JAMA Network Open*.

eFigure 2. Segmented time-series regression analysis for use of ambulance

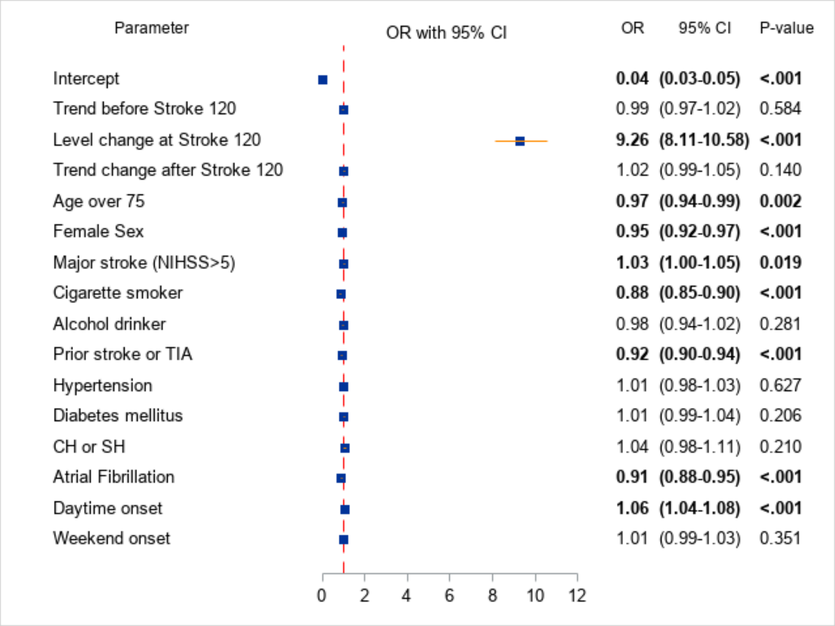

© 2022 Yuan J et al. *JAMA Network Open*.

eFigure 3 Sensitivity analysis 1: Time to hospital and ambulance use per month among all stroke patients (regardless of NIHSS score)

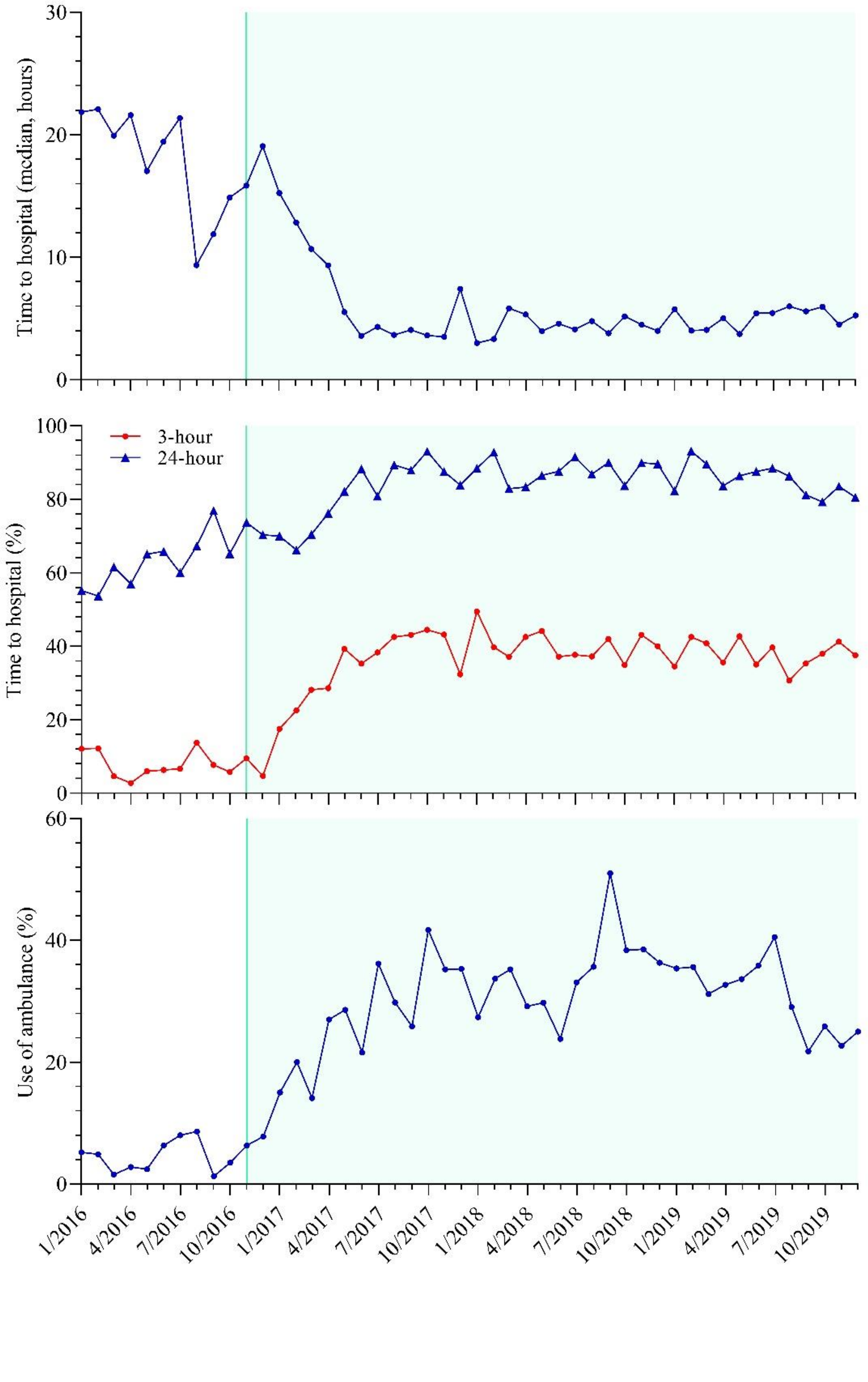

© 2022 Yuan J et al. *JAMA Network Open*.

eFigure 4. Sensitivity analysis 2: Time to hospital and ambulance use per month with a 7-day time window for sample selection

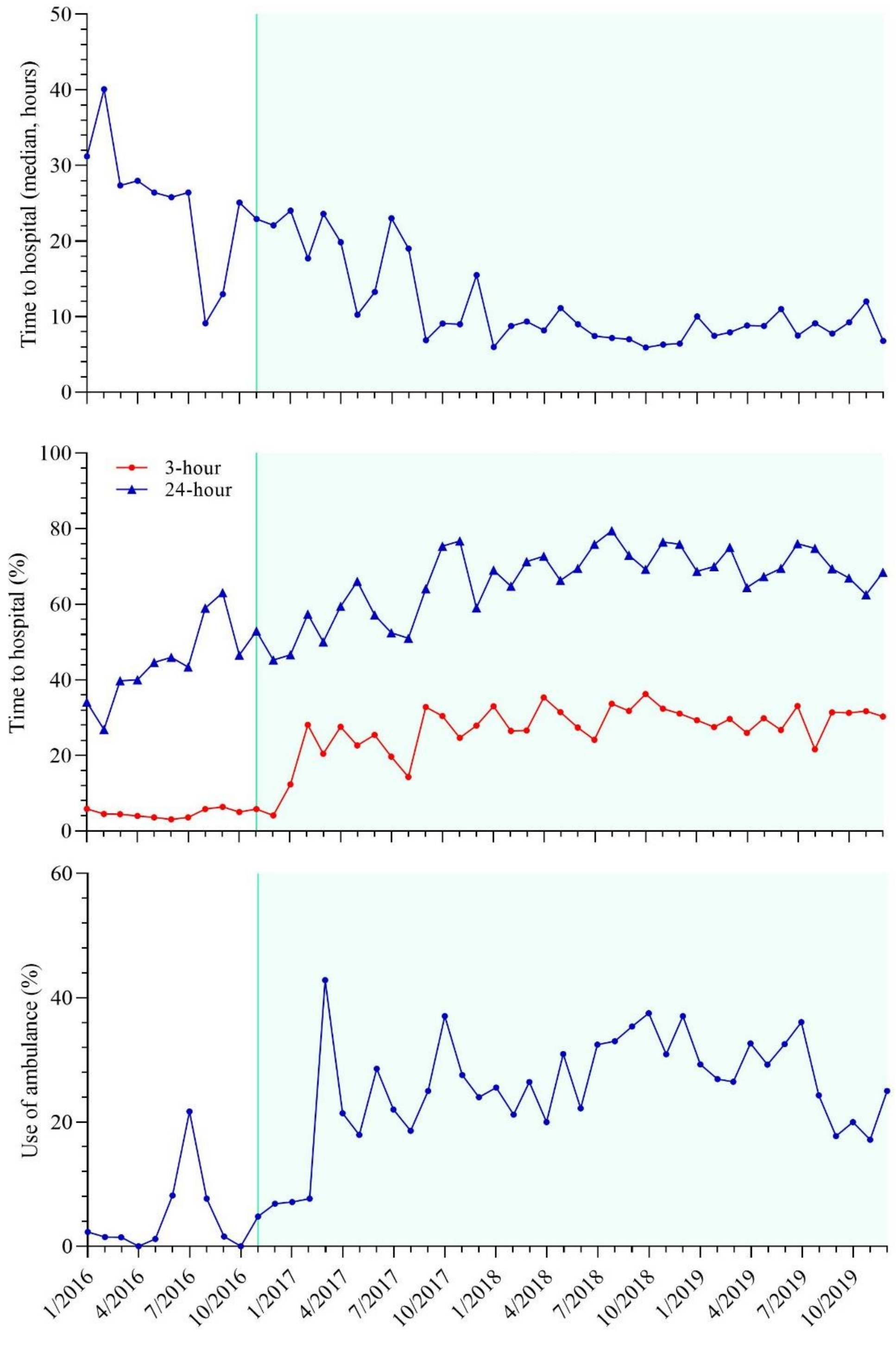

© 2022 Yuan J et al. *JAMA Network Open*.
